# Environmental sustainability in US dairy farms: Policies, practices, and outcomes

**DOI:** 10.1002/jeq2.70031

**Published:** 2025-05-07

**Authors:** Mara L. Cloutier, Daniel Liptzin, Adolfo Coyotl, Abigail E. Baxter, Cristine L. S. Morgan

**Affiliations:** ^1^ Soil Health Institute Morrisville North Carolina USA; ^2^ Department of Land, Air and Water Resources University of California Davis California USA; ^3^ Wilbur‐Ellis Agribusiness Pasco Washington USA

## Abstract

Improving sustainability on US dairy farms has become a critical focus across the industry. As dairy farms continue to consolidate, there is a growing need to identify scalable, implementable soil health management practices that enhance environmental sustainability in the fields managed by the dairy. This paper examines the constraints on dairy forage operations, summarizes key findings from research station experiments comparing soil health management practices in these systems, and synthesizes findings from on‐farm research projects that track environmental outcomes after practice adoption. We discuss the knowledge gaps related to soil health management practices and forage production, highlighting the need for long‐term, actionable research that is applicable to the diversity of dairy operations across the United States. To drive meaningful improvements in environmental sustainability, it is crucial to integrate region‐specific soil health practices, supported by technical and financial support. We conclude that the current body of literature is not adequate to support the widespread adoption of locally appropriate practices, underscoring the urgent need for comprehensive research and support systems to ensure the environmental and economic sustainability of the US dairy industry. Finally, we propose future research directions to address the knowledge gaps and region‐specific challenges through an integrated systems approach, focusing on the farm‐scale impacts of soil health practices across diverse climates and production systems.

## Core Ideas

- Most dairy farms in the United States operate as confined systems, where forages are mechanically harvested for feed.
- Soil health management may improve environmental sustainability in dairy forage production.
- Only four greenhouse gases and eight water quality studies document soil health management on dairy farms.
- Optimal forage production systems must efficiently use resources and maintain economics.
- Future research should focus on a systems approach at the farm scale.

**Abbreviations**
CAFO: Concentrated Animal Feeding Operation
GHG: greenhouse gas
OM: organic matter
SOC: soil organic carbon

## 1 INTRODUCTION

Dairy farms in the United States are complex operations that are mostly classified as confined operations, in contrast to the pasture‐based dairies common in other countries. As of 2014, only 6% of US dairy farms were categorized as grazing operations (USDA APHIS, 2016). Therefore, we focus on confinement‐based dairies, as they represent the majority of dairy farm types. More specifically, the emphasis is on the forage production component of confinement‐based farms, rather than dairy cow or manure management.

Forage production on confinement‐based farms differs from that on grain‐based operations in several ways. Forage crops are harvested down to the stubble, leaving very little residue on the soil. Additionally, the heavy equipment used for harvesting and manure spreading contributes to soil compaction, and manure is typically applied to the fields one or more times per year. Finally, forage production may include perennials or double‐ or triple‐crop rotations, depending on water availability and climate.

Because of their designation as confinement operations, dairy farms operate within an economic and policy framework that imposes national‐ and state‐level constraints on each of these components. There is growing recognition across the dairy sector that improving environmental outcomes related to soil, water quality, water use efficiency, and greenhouse gas (GHG) emissions from dairy forage production needs to be prioritized. These outcomes are necessary to adhere to regulations, maintain a social license to operate, contribute to corporate sustainability goals, and maintain dairy farming as a productive and profitable venture for future generations.

## 2 BACKGROUND ON THE US DAIRY INDUSTRY

**FIGURE 1:**
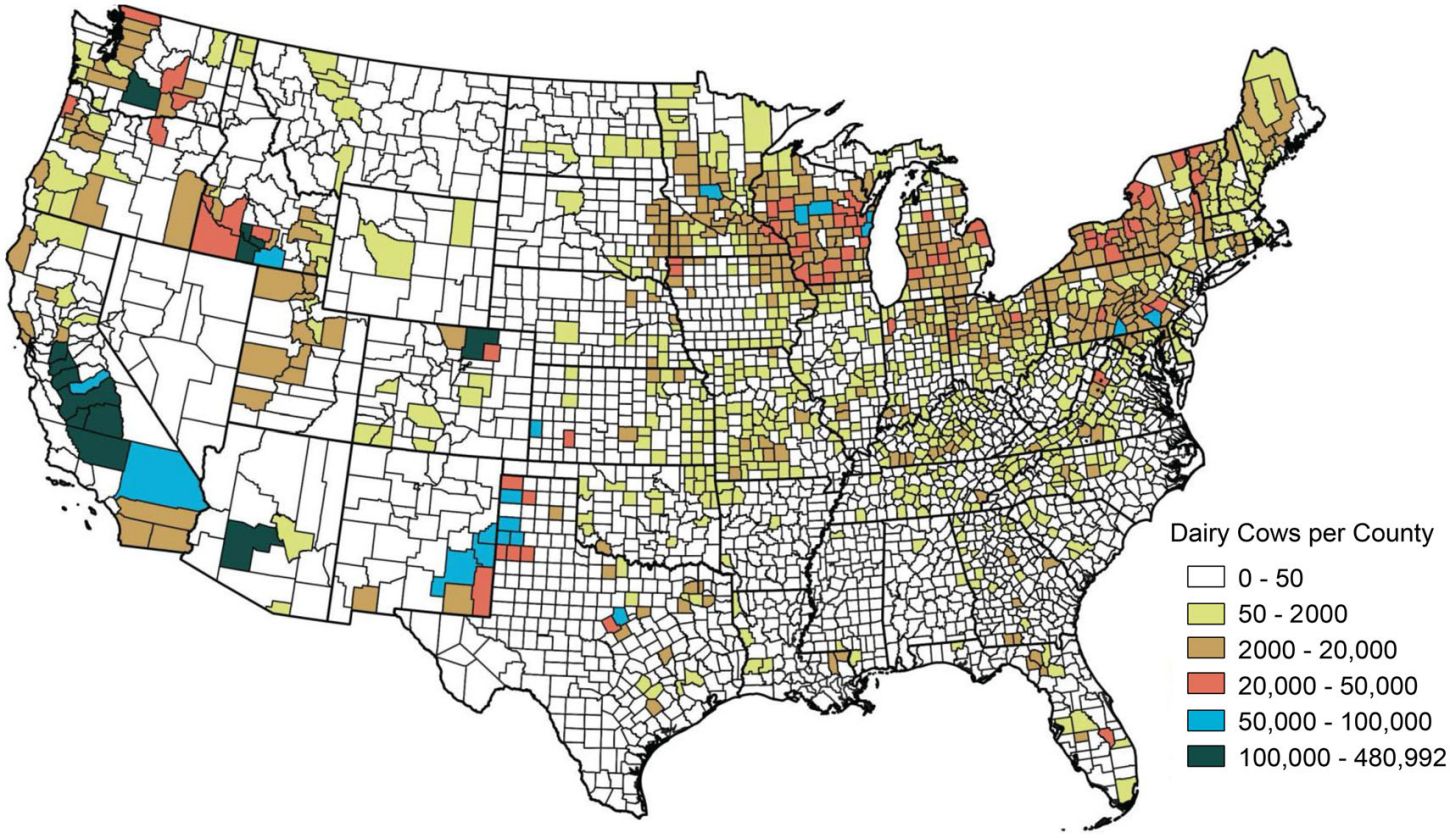
County‐level concentration of milking cows based on the USDA NASS Census of Agriculture 2022 National Level Data.

**FIGURE 2:**
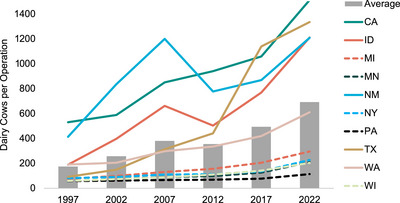
Number of milking cows per farm in the top 10 milk‐producing states in 2022. An average value of milking cows per farm operation is also estimated based on data from the 10 states. Data presented are from the 1997, 2002, 2007, 2012, 2017, and 2022 USDA NASS databases.

Over the past two decades, the US dairy industry has shifted away from smaller dairies characteristic of the Northeast and upper Midwest toward fewer, larger dairy operations typical of the regions in the West and Southwest (Benson, 2008; Khanal et al., 2010; Njuki, 2022; see Figure 1 for dairy cow distribution numbers across the United States). Over a 20‐year period, 2002–2022, the number of dairy operations reduced by 40%. Meanwhile, the number of operations with more than 1000 milk cows increased by 62% (USDA NASS, 2022; see Figure 2 for state‐level information).

As larger dairy operations become more prominent and stocking rates increase, on‐farm forage production to satisfy the nutritional needs of the dairy herd will continue to be strained, further increasing a farm's reliance on outside feed sources (N. P. Martin et al., 2017). Forage acreage associated with dairy farms in the United States is not tracked, but two common crops grown on dairies, silage corn and alfalfa, were harvested on almost 6 and 17 million acres, respectively, in 2022 (USDA NASS, 2022). Expansion of dairy operations to increase milk production results in fewer individuals managing larger areas of land and a more concentrated accumulation of dairy manure on fewer farms. Consequently, growing forage and utilizing manure efficiently to maximize yield and reduce negative environmental outcomes becomes more logistically complex on larger operations, especially in regions with soil and water conservation challenges.

**Core Ideas**

- Most dairy farms in the United States operate as confined systems, where forages are mechanically harvested for feed.
- Soil health management may improve environmental sustainability in dairy forage production.
- Only four greenhouse gases and eight water quality studies document soil health management on dairy farms.
- Optimal forage production systems must efficiently use resources and maintain economics.
- Future research should focus on a systems approach at the farm scale.

## 3 REGIONAL DIFFERENCES IN DAIRY FARMS

Dairy operations differ in their feeding strategies, herd sizes, housing facilities, and manure storage systems (A. Rotz et al., 2021). While these characteristics may differ within a region or state, the greatest differences in these features are found between humid regions (Northeast and upper Midwest) and the semiarid and arid (e.g., irrigated) West and Southwest (Holly et al., 2018). Dairies in humid climates rely on precipitation for crop growth and typically have closed barn operations, yielding liquid dairy manure. Conversely, dairies in the West and Southwest depend heavily on irrigation for forage production, often using dry‐lot confinement systems that produce both liquid manure and dry solids (Holly et al., 2018). These dry‐lot confinement systems may grow two to three forages each year, depending on water availability. Decisions made by farm owners, particularly about herd size, cow housing, and manure management systems, constrain decisions related to forage production. These decisions, influenced by water and land availability as well as the yield needed for cow feed, underscore the unique challenges dairy operations face in supporting milk production and sustainability objectives.

## 4 ECONOMIC AND POLICY LANDSCAPE FOR US DAIRY OPERATIONS

Policy related to environmental outcomes has shaped where and how dairy farms operate in the United States (Sneeringer, S., 2011; Stirm & St‐Pierre, 2003). While most agricultural operations are exempt from the Clean Water Act, Concentrated Animal Feeding Operations (CAFOs), including most dairies, are not exempt (U.S. Congress, 1974; USEPA, 1999). These facilities are required to have permits under the National Pollutant Discharge Elimination System (NPDES) program to manage water pollution. While the Clean Water Act is federal, the NPDES permitting process is implemented by states. The states have wide discretion in implementation, including merging the process with additional state regulations (USEPA, 2002).

In theory, the Clean Air Act, Comprehensive Environmental Response, Compensation, and Liability Act (CERCLA, or Superfund, 42 U.S.C. §§9601–9675), and the Emergency Planning and Community Right‐to‐Know Act (EPCRA, 42 U.S.C. §§11001–11050) all could regulate air emissions from CAFOs, but in practice, these facilities have been considered too small to regulate for criteria pollutants, namely, ammonia and nitrogen oxides. The agricultural sector is largely exempted from the mandatory reporting of GHG emissions, but the U.S. EPA does still estimate emissions from agriculture (USEPA, 2014; 2022).

Additional regulations exist at the state level, but the scope and stringency of the regulations vary widely across states. Several state‐level policies address different components of environmental outcomes. For example, California's Sustainable Groundwater Management Act regulates agricultural water use and requires that farms in certain areas participate in groundwater management programs (Department of Water Resources, California, 2014). Vermont's Required Agricultural Practices includes standards for maintaining buffer zones to reduce pollution of bodies of water (Vermont Agency of Agriculture, Food & Markets, 2016). Wisconsin has restrictions on applying manure in the winter to protect water quality (Wisconsin Department of Natural Resources [WDNR], 2020). Select states also regulate air emissions more than the federal government. For example, since 2003, when California removed its exemption for agricultural sources, many dairy facilities are now regulated by the regional Air Quality Monitoring Districts.

Federal and state assistance programs exist to encourage farmers to adopt practices to improve environmental outcomes. Federal programs such as the Environmental Quality Incentives Program provide both financial and technical aid for conservation practice adoption, including developing a Comprehensive Nutrient Management Plan to improve the storage and application of manure (USDA NRCS, 2018). Many states provide cost‐share incentives and technical support through extension programs and conservation agencies. In efforts to understand the benefit of a cover crop cost‐share programs, researchers have estimated that 54%–98% of the cover crop acreages planted in Iowa, Ohio, and Maryland were a direct outcome of the programs (Fleming, 2017; Mezzatesta et al., 2013; Sawadgo & Plastina, 2021).

## 5 ENVIRONMENTAL SUSTAINABILITY FOR DAIRY OPERATIONS IN THE UNITED STATES

Sustainability goals for dairy operations vary by state, region, and farm. Environmental sustainability efforts may focus on reducing GHG emissions (CO_2_, CH_4_, and N_2_O), minimizing nitrogen (N) and phosphorus (P) losses through erosion and leaching, ammonia (NH_3_) losses that impair air and water quality, improving water use efficiency, or building soil carbon (C) (Afshar, 2023). Research has informed these efforts and supported more sustainable dairy forage production systems. Notably, studies have explored nutrient management for farm‐level planning (Berlingeri et al., 2021; Meyer & Schwankl, 2000; C. M. Miller et al., 2017; Ros et al., 2023) and broader manureshed approaches (Dell et al., 2022; Spiegal et al., 2022), whole‐farm life cycle assessments (A. Naranjo et al., 2020; A. M. Naranjo et al., 2023; C. A. Rotz et al., 2024), comparisons of grazing‐based and row‐crop dairy systems (Becker et al., 2022; Dietz et al., 2024; Rui et al., 2022), and modeling efforts to estimate practice‐driven changes in environmental outcomes (Greene et al., 2024; Hansen et al., 2021; Li et al., 2012; Mason et al., 2020; C. A. Rotz et al., 2024).

Implementing soil health practices can support sustainability goals (Afshar, 2023). The NRCS defines soil health as “the continued capacity of soil to function as a vital living ecosystem that sustains plants, animals, and humans,” underscoring its role in farm efficiency and productivity. Healthy soils improve water and nutrient cycling, enhance climate resilience through greater water holding capacity and infiltration, and increase C storage, contributing to GHG mitigation (as described in the sections below). While numerous indicators exist to assess soil health, such as available water holding capacity, aggregate stability, and microbial activity, this paper focuses on environmental outcomes rather than specific metrics. For further details on soil health indicators, readers can refer to Norris et al. (2020).

Key soil health practices influencing GHG reductions, water quality improvements, and water use efficiency include manure type and application method, tillage, and crop rotations incorporating crop diversity and cover crops. These management decisions affect soil N, P, and C concentrations, field‐scale hydrology, and soil cover. This section highlights soil health practices that can enhance environmental outcomes in dairy forage systems.

### 5.1 Manure type and application method

Environmental outcomes in dairy forage production are significantly influenced by the rate, type, and application method of dairy manure. Research has extensively explored how these factors impact forage yield, N and P losses, and soil organic carbon (SOC) accumulation. There is clear evidence that when manure is applied at the right rates and times, which depend on climate, manure type, soil N and P pools, and other site‐specific variables, it can meet crop nutrient needs while increasing SOC and minimizing N and P losses (Daliparthy et al., 1994; P. L. O'Brien & Hatfield, 2019; Sanderson & Jones, 1997; Stock et al., 2019; R. A. Young & Mutchler, 1976). Optimized manure application can also help minimize salinity issues in arid and semiarid regions by reducing excessive manure inputs (Cabrera et al., 2009). However, our understanding of how manure type and application methods impact the environmental sustainability of dairy forage production remains limited to specific combinations of climate, soil type, and manure type.

Dairy manure varies widely, including slurry, liquid, solids, compost, and liquid digestate. These types differ in terms of moisture content, N and P fractions and concentration, and other properties that influence soil functioning. Manure type does not appear to impact forage biomass or N uptake (Rittl et al., 2023; Saunders, Fortuna, Harrison, Whitefiled, et al., 2012), though inconsistent effects of manure type have been observed for nitrate (NO_3_^−^) leaching and nitrous oxide (N_2_O) emissions (Basso & Ritchie, 2005; Fan et al., 2017; E. C. Martin et al., 2006; Rochette et al., 2008; Saunders, Fortuna, Harrison, Cogger, et al., 2012). Factors such as soil type, application timing, and method influence soil GHG emissions and leaching/runoff from these different manure types. Emissions of N_2_O have been noted to depend on both soil type and weather, with increased emissions in a drier year from a loam soil but more from a clay soil in a wetter year, regardless of manure type applied (Rochette et al., 2008). Timing of applications is also important, as rainfall events shortly following manure application can lead to peak N_2_O emissions (Abalos et al., 2016; Sadeghpour et al., 2018).

Application methods depend on the type of manure that is produced on the farm. Broadcast applications are suitable for applying both dry and liquid manures, while subsurface injection and fertigation methods are useful for liquid manures. Increased N_2_O emissions have been measured from injection practices compared to broadcast in no‐till systems (Duncan et al., 2017; Ponce de León et al., 2021; J. Sherman et al., 2021), with potential differences in N_2_O emissions dependent on weather conditions, whereby wetter, but not fully saturated, soils emit more (Sadeghpour et al., 2018). Similarly, injecting manure and incorporating manure using tillage often leads to increased NO_3_^−^ leaching compared to surface applications in no‐till systems (Dell et al., 2011; Gupta et al., 2004; Powell et al., 2011). While subsurface applications of liquid manures seem to have more negative environmental consequences in humid regions, this method of application is presumably a better alternative when flood irrigation is employed (Burger et al., 2016). The emissions of N_2_O and leaching of NO_3_^−^ are highly variable in response to manure type and application; however, it is well established that injecting slurry or rapidly incorporating solid manure are highly effective ways to reduce NH_3_ emissions (Webb et al., 2010).

### 5.2 Reduced tillage

Dairy forage operations use tillage to control weeds, incorporate manure, terminate perennial crops and cover crops, treat soil compaction, and create a smooth seedbed. However, studies have demonstrated that tillage is not necessary to maintain a high‐yielding, high‐quality forage operation (Amin et al., 2018; Kaneko et al., 2011; Reed et al., 2019). More generally, in annual cropping systems, reduced tillage results in positive soil health outcomes (Bagnall et al., 2022; Liptzin et al., 2022), including soil aggregation, which is a component of soil structure (Rieke et al., 2022). Improved soil structure can decrease runoff and erosion, resulting in better water quality and more efficient nutrient use (Basset et al., 2023; Zhang et al., 2007). Reducing tillage also enhances water use efficiency and water holding capacity (Hatfield et al., 2001; Zibilske & Bradford, 2007).

Empirical evidence for how tillage reductions impact GHG emissions in manured forage operations provides mixed results that may have an important temporal component. Some studies have found increased N_2_O emissions under no‐till, while others have measured fewer emissions in no‐till compared to plow or disk tillage treatments (Badagliacca et al., 2018a, 2018b; Baggs et al., 2003; Gregorich et al., 2008; Huang et al., 2015; Omonode et al., 2011; Plaza‐Bonilla et al., 2014; Six et al., 2004; Tan et al., 2009; Tellez‐Rio et al., 2015; Ussiri et al., 2009). These contrasting results may be due to the number of years since no‐till or reduced‐till management was established (Plaza‐Bonilla et al., 2014; van Kessel et al., 2013). A meta‐analysis found that N_2_O emissions were greater in no‐till/reduced‐tillage treatments compared to conventionally tilled treatments until after 10 years of implementation (van Kessel et al., 2013).

Another factor influencing the relationship between GHG emissions and tillage intensity is soil texture. For example, Pelster et al. (2021) observed greater N_2_O emissions from reduced tillage compared to conventional tillage on clay soil, while no differences were observed on sandy loam soil. Soil texture may be a key predictor of environmental outcomes with tillage practices (Ekwunife et al., 2022; Mhazo et al., 2016; Sedghi et al., 2024; Sun et al., 2015) due in part to the relationship with available water holding capacity (Saxton et al., 1986; van Es et al., 1999).

### 5.3 Diversifying crop rotations

Forage rotations that include perennials, double crops, or cover crops can also influence sustainability outcomes compared to annual‐annual rotations (N. P. Martin et al., 2017). Including a perennial forage can offset soil C losses, GHG emissions, soil erosion, and nutrient runoff during the annual years of the rotation (Gamble et al., 2022; Maas et al., 2013). Cover crops offer similar benefits (Faé et al., 2009; Schipanski et al., 2014). Diverse forage crop rotations can also enhance soil health by improving aggregate stability, increasing microbial activity, and increasing SOC and forage production (Acharya et al., 2024; Entz et al., 2002; Halvorson et al., 2002; W. E. Jokela et al., 2009; Maiga et al., 2019; Sanford et al., 2021; Zaeem et al., 2019). Multi‐year growth of perennial forages in a rotation can lead to greater belowground biomass and deeper rooting depths compared to annual–annual rotations. Perennial forage roots can remain active year‐round and for years after transitioning back to annual crops (Dupont et al., 2014; Fan et al., 2016; Lasisi et al., 2018; Monti & Zatta, 2009).

Despite the potential benefits of including perennials or cover crops in forage rotations, the method of transitioning them to annual crops is very important. Several studies have measured peak GHG emissions following residue incorporation via spring tillage, a consequence that is exacerbated if N is also applied in the spring (Abalos et al., 2016; Adelekun et al., 2019; Baggs et al., 2000; Basalirwa et al., 2020; Kandel et al., 2020; Tenuta et al., 2019; Trozzo et al., 2020). Furthermore, terminating alfalfa with tillage increases N mineralization rates as evidenced by increased soil inorganic N supplies compared to alfalfa terminated with herbicide and left on the soil surface to decompose (Malhi et al., 2010; Mohr et al., 1999, 1998). Retaining perennial or cover crop residue on the soil surface, rather than incorporating it with tillage, is crucial to maintain environmental benefits from these diverse crop rotations.

Double‐crop rotations provide an opportunity to maintain soil cover during winter months while increasing total forage yield. These rotations have become the prevailing management in some regions (Dell et al., 2024) in areas with sufficient water availability and growing degree days, triple‐crop rotations are possible. The distinction between cover cropping and double cropping often comes down to whether the crop is harvested, as many cover crops can also serve as forage. Double cropping is more feasible in warmer regions, due to the increased number of growing degree days. Several studies have demonstrated the implementation potential of winter forages in colder climates (Jemison et al., 2012; J. R. West et al., 2020). This practice offers benefits for nutrient management (Baxter et al., 2023; Binder et al., 2020; Krueger et al., 2012; Newton et al., 2003) and water storage (Yost et al., 2023).

In a survey of dairy farmers in New York who were double cropping, researchers found that farmers were only planting about 8% of their row crop fields with a winter cereal (Ketterings et al., 2015). The ability to increase total forage yield through double cropping may encourage farmers to invest in planting a crop in the fall, but the economics are not always favorable (Ranck et al., 2019). Identifying the most suitable double crop options to meet producer needs, particularly in colder regions, is an emerging area of interest (Liebert et al., 2023).

### 5.4 Multiple practices and managing forage production as a system

Implementing a single soil health management practice can result in positive changes toward environmental sustainability, as described in the previous sections. Studies have found that combining practices or implementing a new system have the best overall improvements to environmental outcomes (P. L. O'Brien et al., 2022). Three examples of how combining soil health management practices can improve environmental outcomes are briefly described below.

Incorporating a perennial crop such as alfalfa into a forage rotation leads to increased soil C and reduced GHG compared to annual–annual forage rotations (Maas et al., 2013). However, terminating the alfalfa with tillage results in significant losses of GHG, ultimately offsetting the conserved GHG emissions during the alfalfa years of the rotation (Tenuta et al., 2019). Another example of how integrating multiple practices could help negate unintended consequences is using winter crops, either cover crops or double crops, to capture available soil N and reduce leaching from no‐till fields (Nouri et al., 2022). As no‐till improves soil structure and porosity, drainage improves and leaching potential increases compared to tilled soils (Li et al., 2023). Conversely, pairing no‐till with cover cropping enhances soil water retention potentially due to increased soil organic matter (Nunes et al., 2018; Villamil et al., 2006). This is particularly important in arid and semiarid regions, where cover cropping alone can reduce available water for spring planted crops (Mitchell et al., 2015; Nielsen et al., 2016; J. Wang et al., 2021).

Identifying systems that support goals of optimizing yield and forage quality through improved nutrient use efficiency and water cycling should improve environmental outcomes. In a recent study, researchers identified the optimum system for forage production and soil health in Vermont to be no‐till with cover crops and a 6‐year corn and 4‐year alfalfa rotation (White et al., 2023). Of course, these practices or systems need to be tailored for individual farms to account for differences in soil texture, topography, water availability, climate, forage production needs, and other factors.

## 6 WHAT DO WE KNOW ABOUT ENVIRONMENTAL OUTCOMES ON COMMERCIAL US DAIRY FARMS?

While plot‐scale and field‐scale studies of forage production on fields not historically associated with dairies can provide valuable information, the differences in the equipment used and the lack of a history of manure are limitations for extrapolating results to the fields of commercial dairy farms. Our ability to implement soil health practices on‐farm and relate these practices to changes in environmental outcomes is imperative. On‐farm research presents several challenges, including variations in soil types, adaptations to management practices that are requested by farmers, the lack of controlled environment necessary for certain research objectives, and the associated costs (Ellis & Paustian, 2024; Karlen et al., 2017; Krueger et al., 2013).

Despite these challenges, on‐farm studies with management relevant to commercial dairy operations are essential for assessing whether outcomes from long‐term agricultural experiments hold under real‐world conditions for dairy forage production. On‐farm studies generate data for calibrating and validating process‐based biophysical models (Ellis & Paustian, 2024), foster innovation between researchers and producers (Lacoste et al., 2022), and build confidence among farmers by demonstrating the effectiveness of practices in practical applications (Pires et al., 2024). Below, we highlight environmental outcomes of soil health management practices based on peer‐reviewed research conducted on commercial US dairy operations.

### 6.1 Soil carbon

Increasing SOC on farms has become a focal point in efforts to mitigate GHG footprints and address climate change. Comparative studies across cropping systems, including both dairy and non‐dairy operations, highlight that perennial grasses and pastures exhibit greater SOC or organic matter (OM) in the top 0–15 cm of soil compared to row‐crop operations (Augarten et al., 2023; Lei et al., 2021; Nunes et al., 2020). Specifically, when comparing row crop systems predominantly in the Northeast, dairy systems showed increased SOC compared to single vegetable, fruit, and grain cropping systems but lower SOC than perennial and mixed‐vegetable systems (Nunes et al., 2020). However, a Wisconsin‐based study by Augarten et al. (2023) found no differences in OM between annual‐perennial dairy fields and annual‐only rotations with or without manure. Considering that these were cropping systems comparisons, mostly driven by cash crop rotation and manure amendments, it is unclear what impact cover crops, double crops, or reduced/no‐till had on the findings.

Research exploring the impact of management practices such as tillage, cover cropping, or applying alternative manure types on SOC on commercial US dairy farms remains limited. From a study comparing C pools on Pennsylvania dairy farms, the evidence suggests that no‐till management enhances SOC storage in the top 0–15 cm compared to conventional tillage practices, with potential annual accumulations estimated at approximately 0.5 Mg C ha^−1^ (Dell et al., 2008). Other studies have identified SOC increases by combining manure with biochar applications or integrating cover crops (Angst et al., 2014; Krueger et al., 2013). Moreover, correlations have been observed between OM and the stability of forage yield on a farm in New York (E. A. Long & Ketterings, 2016), indicating broader implications for soil health and productivity. Despite the limitations in geographic representation across these studies and the number of farms represented, the findings underscore the potential of soil health management practices to enhance C sequestration on commercial dairy farms and improve agricultural resilience.

### 6.2 Nitrous oxide emissions

A comprehensive assessment of GHG emissions on a commercial dairy farm would include emissions from the cows, manure management, and soil, as well as fuel and energy use (Rotz et al., 2021). Among these, soil‐based emissions are primarily driven by N_2_O emissions associated with dairy manure applications. We reviewed the published literature on N_2_O emissions associated with dairy manure applications to commercial croplands in the United States and summarized the characteristics of the 28 experiments in Table 1. Statistical comparison of these studies is challenging because of the variability in climate, manure type (solid vs. liquid, raw vs. processed), and application method (broadcast vs. injected) used. The average study duration was 2 years, but fewer than half of the studies measured emissions year‐round.

**TABLE 1:** Studies from commercial dairies and research stations in the United States to assess greenhouse gas (GHG) emissions from soils fertilized with dairy manure and used to grow forage.

State	Farm type	Length of data collection	GHGs quantified	Soil health practices	Fertility practices	Reference
California	Commercial dairy	1 Year	CO_2_, CH_4_, N_2_O	Organic type	Nutrient source	Gao et al., 2023
California	Commercial dairy	1 Year	CH4, N_2_O	Organic type	None	Angst et al., 2014
California	Commercial dairy	1.5 Years	CO_2_, N_2_O	None	Nutrient source	Lazcano et al., 2016
California	Commercial dairy	1.8 Years	CO_2_, CH_4_, N_2_O	None	None	Owen & Silver, 2017
Colorado	Research station	3 Years	CO_2_, CH_4_, N_2_O	Organic type	Nutrient source	Halvorson et al., 2016
Idaho	Research station	3 Years	CO_2_, CH_4_, N_2_O	Organic type	Timing, nutrient source	Dungan et al., 2017
Idaho	Research station	3 Years	CO_2_, CH_4_, N_2_O	Organic type	Timing, nutrient source	Dungan et al., 2021
Idaho	Unclear	3 Years	CO_2_, CH_4_, N_2_O	Organic type	Nutrient source	Lentz et al., 2014
Idaho	Research station	4 Years	CO_2_, N_2_O	None	Rates, nutrient source	Leytem et al., 2019
Idaho	Research station	2 Years	N_2_O	None	Rates, nutrient source	Dungan et al., 2023
Idaho	Research station	3 Years	CO_2_, CH_4_, N_2_O	Organic type	Rates, nutrient source	Dungan et al., 2021
Michigan	Research station	2 Years	CH_4_, N_2_O	Organic type	Nutrient source	Thelen et al., 2010
New Hampshire, Maine, Vermont	Research stations and commercial dairy	2 Years	CO_2_, N_2_O	Organic type	Nutrient source	Contosta et al., 2021
New York	NA	2 Years	N_2_O	Crop type	Placement	Sadeghpour et al., 2018
New York	Commercial dairy	2 Years	N_2_O	None	None	Molodovskaya et al., 2012
New York	NA	2 Years	N_2_O	Organic type, tillage	Rate, nutrient source	Sadeghpour et al., 2017
New York	Commercial dairy	1 Year	N_2_O	None	Timing, placement	Singurindy et al., 2009
Pennsylvania	Research station	2 Years	N_2_O	None	Placement	Duncan et al., 2017
Pennsylvania	Research station	2 Years	N_2_O	Crop type	Nutrient source, placement	Ponce de Leon et al., 2021
Pennsylvania	Research station	2 Years	CO_2_, N_2_O	Crop rotation	Nutrient source	Adviento‐Borbe et al., 2010
South Dakota	NA	2 Years	CO_2_, CH_4_, N_2_O	Organic type	Nutrient source	Abagandura et al., 2019
South Dakota	Research station	2 Years	CO_2_, CH_4_, N_2_O	None	Rates, nutrient source	Ozlu & Kumar, 2018
Vermont	Research station	3 Years	CO_2_, N_2_O	Tillage	Placement	Dittmer et al., 2020
Washington	Commercial dairy	2 Years	CO_2_, CH_4_, N_2_O	Organic type	Nutrient source	Collins et al., 2011
Wisconsin	Research station	2 Years	CH_4_, N_2_O	Crop rotation, tillage	Rates, nutrient source, placement	Osterholz et al., 2014
Wisconsin	Research station	3 Years	CO_2_, CH_4_, N_2_O	Tillage	Placement	J. Sherman et al., 2021
Wisconsin	Research station	2 Years	CO_2_, CH_4_, N_2_O	Tillage	Placement	Sherman & Young, 2022
Wisconsin	Research station	1 Year	CO_2_, CH_4_, N_2_O	Organic type	None	Holly et al., 2017

Abbreviation: NA, not available.

Out of the 28 studies presented in Table 1, only eight assess changes to GHG emissions from on‐farm experiments. From these eight studies, the importance of nutrient management, optimal tillage timing, and the application of biochar were highlighted as effective strategies for reducing GHG emissions (Angst et al., 2014; Collins et al., 2011; Gao et al., 2023; Lazcano et al., 2016; Molodoyskaya et al., 2012; Singurindy et al., 2009). These studies do not provide sufficient scientific evidence regarding the broader impact of soil health management systems on GHG emissions from US dairy farms. Additional research is needed in different regions beyond those studied (California, New York, Washington, and Vermont) to better reflect the diversity of soils, climates, and cropping systems that US dairy encompasses. Moreover, other practices need to be explored including cropping rotation diversity, various tillage practices, and different methods for nutrient application.

### 6.3 Water quality

Export of N and P from forage production fields through leaching and runoff contribute to reduced water quality. Mass balances and nutrient management plans can be effective approaches to evaluate opportunities to better optimize N and P use on dairy farms (Cela et al., 2015; Ghebremichael & Watzin, 2011; Olivo et al., 2024; Ros et al., 2023; Soberon et al., 2015), ultimately reducing losses to water sources. However, studies comparing the effectiveness of various conservation practice (e.g., reduced tillage, cover cropping, and alternative manure types) on water quality indicators from commercial US dairy operations are limited. Table 2 provides an overview of existing water quality studies on fields that received dairy manure, including those conducted at university or USDA research sites that were historically in dairy forage production. Of the 23 on‐farm studies, 10 compared soil health management practices, and only five of those 10 measured water quality outcomes for longer than six months.

**TABLE 2:** Studies from commercial dairies and research stations in the U.S. to assess runoff and/or leaching from soils fertilized with dairy manure and used to grow forage.

State	Farm type	Collection method	Length of data collection	N and P pools quantified	Soil health practices	Fertility practices	Reference
NA	NA	Runoff	3 Years	NO_3_^−^, NH_4_^+^	None	Nutrient source	F. L. Long et al., 1975
California	Commercial dairy	Lysimeter	5 Months	NO_3_^−^, NH_4_^+^, Ortho‐P	Organic type	Nutrient source	Gao et al., 2023
California	Commercial dairy	Monitoring well and tile drainage	5 Years	NO_3_^−^	None	None	Van der Schans et al., 2009
California	Commercial dairy	Monitoring well	4 Years	Total N, NO_3_^−^	None	None	Harter et al., 2002
California	Commercial dairy	Monitoring well	7 Years	NO_3_^−^	None	Rates	Harter et al., 2001
California	Commercial dairy	Monitoring well	Unclear	NO_3_^−^	None	None	C. M. Miller et al., 2020
Florida	Commercial dairy	Lysimeter	4 Years	NO_3_^−^	Crop rotation	Rates	Woodard et al., 2002
Florida	Commercial dairy	Lysimeter	4 Years	NO_3_^−^	Crop rotation	Rates	Woodard et al., 2003
Florida	Commercial dairy	Monitoring well	7 Years	DRP	None	None	Duan et al., 2012
Florida	Commercial dairy	Lysimeter	3 Years	Total P, DRP	None	Rates	Woodard et al., 2007
Idaho	NA	Runoff	2 Years	NO_3_^−^, NH_4_^+^, Total P, DRP	None	Nutrient source	Lentz & Lehrsch, 2010
Michigan	NA	Subsurface drainage	5 Months	Total N, NO_3_^−^, NO_2_^‐^, Total P, Ortho‐P	Tillage	Rate, placement	Haack & Duris, 2008
Minnesota	Research station	Runoff	3 Years	Total N, NO_3_^−^, NH_4_^+^, Total P, Ortho‐P	Crop type, tillage	Timing, placement	R. A. Young & Mutchler, 1976
Minnesota	Commercial dairy	Runoff and subsurface drainage	3 Years	NO_3_^−^, DRP	Crop rotation	None	Krueger et al., 2013
Minnesota	Commercial dairy	Subsurface drainage	2 Years calibration, 3 years of treatments	NO_3_^−^, DRP	Crop rotation	None	Gamble et al., 2022
Minnesota and Wisconsin	Research stations	Runoff	6 Months	Total N, NH_4_^+^, total P, DRP, particulate P	None	Rates	E. Young et al., 2022
New York	Commercial dairy	Runoff	1 Year	Total P, DRP	Crop rotation	None	Kleinman et al., 2005
New York	Commercial dairy	Stream collections	1 Year	DRP	None	Rate, placement, timing	Easton et al., 2008
New York	Research station	Runoff and tile drainage	15 Months	Total N, NO_3_^−^, total P, DRP, particulate P	Crop rotation	None	Griffith et al., 2020
New York	Research station	Runoff	3 Years	NO_3_^−^, NH_4_^+^, inorganic P, total soluble P	None	Rates	Klausner et al., 1976
New York	Commercial dairy	Piezometer	4 Years	NO_3_^−^, DRP	None	Rates	Flores‐López et al., 2011
New York	Commercial dairy	Runoff simulations	Two time points	Total P, particulate P, DRP,	None	None	Hively et al., 2005
New York	Research station	Monitoring well	4 Years, 3 years	Total N, NO_3_^−^	None	None	S. J. Wang et al., 1999
New York and Wisconsin	Commercial dairy	Runoff and tile drainage	3 Years	Total N, NO_3_^−^, NO_2_^‐^, NH_4_^+^, Total P, Ortho‐P	None	Nutrient source	Fermanich et al., 2023
OH	Commercial dairy	Runoff and subsurface drainage	Unclear	Total P, DRP	None	Nutrient source	King et al., 2018
Pennsylvania	Commercial dairy	Runoff simulations	Three events	Total P, DRP	Tillage	None	Garcia et al., 2008
Pennsylvania	Research station	Runoff and runoff simulations	2 Years	Total P, DRP	Tillage	Placement	Veith et al., 2011
Pennsylvania	Commercial dairy	Runoff simulations	Two time points	Total P, DRP	Tillage	None	Kleinman et al., 2009
Pennsylvania	Research station	Runoff, subsurface drainage	5 Years	Total P, DRP, particulate P	None	Placement	M. D. Miller et al., 2019
Pennsylvania	Research station	Lysimeter	4 Years	Total P, DRP, particulate P	None	Placement	Jahanzad et al., 2019
Pennsylvania	Research station	Runoff simulations	2 Years	Total P, DRP	Crop rotation, tillage	None	Verbee et al., 2010
Pennsylvania	Research station	Runoff	14 Months	Particulate P and DRP	Organic type	None	Vadas et al., 2007
Pennsylvania	NA	Lysimeter	4 Years	Total P, NO_3_^−^	Crop rotation	Rate, nutrient source	Toth et al., 2006
Vermont	NA	Runoff & Lysimeter	5.5 Years	Total N, total P, DRP, and dissolved P	None	Placement	Twombly et al., 2021
Vermont	Commercial dairy	Runoff	5 Years	Total P	None	None	Kominami & Lovell, 2012
Wisconsin	Commercial dairy	Runoff	4 Years	Total N, NO_3_^−^, NO_2_^−^, NH_4_^+^, total P, DRP	None	Rate, timing, nutrient source	Komiskey et al., 2011
Wisconsin	NA	Runoff	2 Years	Total P, DRP, algal‐available P	Tillage	None	Mueller et al., 1984
Wisconsin	Research station	Runoff simulations	Four events	Total P, DRP,	None	Timing	Grande et al., 2005
Wisconsin	Research station	Runoff simulations	Three events	Total N, NO_3_^−^, NH_4_^+^, Total P, DRP, particulate P	Tillage	Placement	W. Jokela et al., 2016
Wisconsin	Research station	Runoff	1 Year	Total P, DRP, bioavailable P	Organic type	None	Ebeling et al., 2002
Wisconsin	Research station	Runoff	1 Year	Total N, NO_3_^−^, NH_4_^+^, total P, DRP	Grassed waterways, field borders	Timing, placement	W. Jokela & Casler, 2011
Wisconsin	NA	Runoff simulations	1 Month	Total P, DRP	Crop rotation	None	Grabber & Jokela, 2013
Wisconsin	Research station	Runoff	4.5 Years	Total N, NO_3_^−^, NH_4_^+^, total P, DRP	Crop rotation, grazing	None	Vadas & Powell, 2013
Wisconsin	Research station	Runoff simulations	Four events	Total N, NO_3_^−^, NH_4_^+^, total P, DRP	Cover crop, buffer strip	Timing	J. Sherman et al., 2021
Wisconsin	Research station	Runoff	2 Years	Total N, NO_3_^−^, NH_4_^+^, total P, DRP	Tillage	Timing	Stock et al., 2019
Wisconsin	Research station	Runoff	2 Years	Total P, DRP	Tillage	Timing	Vadas et al., 2019
Wisconsin	Research station and commercial dairy	Runoff	6 Years	Total P, DRP	None	None	Good et al., 2012
Wisconsin	Research station	Drainage lysimeter	4 Years	NO_3_^−^	Tillage	Placement	Powell et al., 2011
Wisconsin	Research station	Runoff	3 Years calibration, 3 years treatments	Total N, NO_3_^−^, NO_2_^−^, NH_4_^+^, total P, DRP	Crop rotation, tillage	Timing	J. F. Sherman et al., 2020

Abbreviations: DRP, dissolved reactive phosphorus; NA, not available.

Results from two on‐farm studies that compared water quality outcomes from different tillage practices largely aligned with results obtained at research stations. Specifically, incorporating manure into the soil using tillage reduces N and P runoff compared to broadcast applications with no tillage (Garcia et al., 2008; Kleinman et al., 2009; Mueller et al., 1984; Stock et al., 2019; Vadas et al., 2019; R. A. Young & Mutchler, 1976). Low disturbance application methods, such as vertical tillage or shallow disc injection, are effective at reducing N and P losses through runoff (W. Jokela et al., 2016; Powell et al., 2011; Veith et al., 2011) and maintaining soil structure. However, the most effective system for reducing nutrient runoff losses involves broadcasting manure onto a standing cover crop in a no‐till system (Verbee et al., 2010).

On‐farm studies have shown that crop rotations with perennials and cover crops can reduce N and P losses. Krueger et al. (2013) demonstrated that a rye cover crop reduced N leaching by nearly 50% in its first year. Further research on the same farm found significantly lower NO_3_^−^ and P losses in an alfalfa field compared to a corn silage field (Gamble et al., 2022). The utility of perennial crops to reduce nutrient losses to water has also been demonstrated at a commercial farm in Florida (Woodard et al., 2002, 2003). Given the limited scope of these on‐farm studies, both in terms of soil health management practice comparisons and regions represented, there is a need for more comprehensive research to determine which practices lead to the most favorable water quality outcomes for the US dairy industry.

### 6.4 Water quantity

Water use efficiency is becoming increasingly critical for dairy farms, particularly in water‐limited environments in the western United States. The depletion of aquifers, such as the Ogallala Aquifer, which supplies water to the Texas High Plains, has reduced the available water for dairy farms. This has forced some dairy farms to grow forage with little or no irrigation, leading to limitations on the forage varieties grown and overall yields (Bhattarai et al., 2019; Cruz et al., 2021). While the Ogallala Aquifer illustrates prominent water scarcity challenges for dairy farms, similar issues are expected in other regions as climate change continues to disrupt precipitation patterns (Liu et al., 2022). Soil health practices can play a vital role in supporting water conservation efforts in semiarid regions (Nilahyane et al., 2023).

Water availability significantly constrains agronomic potential in arid and semi‐arid regions of the United States, as well as in humid regions during drought periods. Dairy farms in the western United States primarily rely on irrigation to supply their water needs. However, irrigation comes at a considerable financial cost that could be prohibitive for some farmers. Currently, crop irrigation constitutes the largest water use by US dairy farms (Matlock et al., 2013). To adapt to anticipated drought, dairy farmers in some semiarid regions have started growing silage crops that have more tolerance to water stress compared to traditional corn silage (Bhattarai et al., 2019).

Despite the critical need, our review of the literature only uncovered one study evaluating forage water usage on commercial operations in relation to soil health management practices. On a farm in Minnesota, corn silage was found to have a greater water use efficiency than alfalfa (∼70% increase; Gamble et al., 2022). As farmers navigate decisions related to water use for forage production, whether they are facing drought conditions or depleting wells, the economic implications of producing less or possibly no forages cannot be overstated (Leister et al., 2015). Deines et al. (2020) estimated that nearly one in 10 irrigated fields may be unable to support crop production when irrigation is no longer viable. Research focused on scalable and practical strategies for enhancing water use efficiency on dairy farms will be essential for sustaining US milk production, particularly with the ongoing expansion of dairy operations in these water‐limited regions.

### 6.5 Forage productivity

For dairy farms, the quality and yield of forage grown on the farm are crucial economic factors, necessitating an understanding of how soil health management practices impact productivity. A review of the literature identified eight studies examining yield differences in US dairy‐producing fields, with four also exploring forage quality (Battaglia et al., 2021; Cox et al., 2009; Ketterings et al., 2013; Kleinman et al., 2005; Krueger et al., 2013; E. A. Long & Ketterings, 2016; Macoon et al., 2002; Woodard et al., 2002, 2003, 2007). Among these, three studies compared manure rates and forage rotations on dairies in Florida. The findings indicated no significant yield differences based on manure rates, while the rotations with bermudagrass resulted in the highest forage yield (Macoon et al., 2002; Woodard et al., 2002, 2003).

In New York, three on‐farm studies investigated the effects of tillage intensity, tillage depth, and/or manure placement on corn silage yield and quality. Increased yields were observed when manure was incorporated using reduced tillage methods compared to chisel incorporation or no‐till, although no differences in forage quality were observed (Ketterings et al., 2013). When coupling reduced tillage with manure injection, similar corn silage yields and quality were achieved compared to more intensive tillage practices (Battaglia et al., 2020). In a manure injection and reduced tillage system, the depth of tillage impacted silage yield and N use efficiency. Among the tested depths of 0, 18, and 36 cm, the 18‐cm depth appeared optimal for yield, NO_3_^−^ uptake, and profitability (Cox et al., 2009).

Two other studies compared the effect of cover cropping on yield. Krueger et al. (2013) found that corn silage yield decreased by 16% following a winter rye cover crop, primarily due to delayed planting caused by excess moisture on a farm in Minnesota. In contrast, Kleinman et al. (2005) reported a decrease in corn silage yield with interseeded perennial ryegrass but observed no significant yield difference with interseeded red clover compared to no cover crop on a farm in New York. These contrasting outcomes highlight the complexity of cover crop impacts on forage yield, which may depend on the specific cover crop type, local soil conditions, and management practices. Notably, Krueger et al. (2013) emphasized the challenge of late corn planting in winter rye fields due to moisture issues, underscoring the importance of adapting management practices to account for weather. Effective cover crop management, similar to other soil health practices, requires experience (Bagnall et al., 2020). Both the Krueger et al. (2013) and Kleinman et al. (2005) studies only tested 1 year of cover cropping. To comprehensively understand how soil health management practices influence forage yield across varying conditions and over multiple growing seasons, long‐term studies are necessary.

## 7 OUTLOOK FOR US DAIRY FORAGE OPERATIONS

Considering the scarcity of research conducted on dairy farms to evaluate the impacts of management practices on environmental outcomes, it is difficult to estimate when and where soil health management practices will have the biggest impact for the dairy industry. Not only are there few on‐farm studies, but there is a lack of regional representation of dairy forage operations, particularly in arid and semiarid regions of the western United States, included in the current body of literature. Furthermore, many of these on‐farm studies were too short in duration to fully capture changes associated with soil health management, namely, those related to soil structure. It may take 4 or more years to observe improvements in soil health after adopting such practices (W. E. Jokela et al., 2009; Meals et al., 2010; T. O. West & Six, 2007; Wood & Bowman, 2021).

We focused on approaches that can improve outcomes in the forage production fields of confined dairies. At the scale of the whole dairy, modeling studies in humid regions suggest that seasonal or year‐round pasture‐based systems can have lower overall environmental impact than confined systems (Belflower et al., 2012; D. O'Brien et al., 2012; Rotz et al., 2009; Wepking et al., 2022). While it may not be possible in more arid regions, incorporating grazing in the fall or spring may be an option in more humid regions.

Identifying the most effective soil health management practices to reduce negative environmental outcomes and sustain or increase forage yield for a specific farm or region is only the first step toward their adoption. Farmers in some areas may encounter logistical challenges related to the implementation of these practices, which could be mitigated by assistance programs (Carlisle, 2016). Examples of these challenges include the cost or availability of specialized equipment and cover crop seeds, as well as the difficulty of integrating the practice into existing farm operations (Dunn et al., 2016). Even if an optimal dairy forage system is identified, farmers will still benefit from technical assistance to tailor these practices to their specific operations (Bowman et al., 2022). For instance, an on‐farm trial by Krueger et al. (2013) noted that a participating farmer chose not to grow a cover crop during the second year of a 2‐year project because the field with the cover crop was still wet in late spring, causing delayed planting and reduced silage yield during the first year. With access to technical assistance, the farmer might have successfully managed the cover crop to meet their farm goals and continued using cover crops. Similarly, 10 of the 73 dairy farmers surveyed in New York were no longer planting cover crops due to various reasons related to planting dates of both the cover crop and cash crop (Long et al., 2013).

## 8 FUTURE DIRECTION

The trend toward larger, consolidated US dairy operations, particularly in arid and semiarid regions, presents both challenges and opportunities for enhancing environmental sustainability. This paper explored how locally appropriate soil health management practices can mitigate negative environmental outcomes associated with dairy forage production, including GHG emissions, nutrient leaching, runoff, and water use. However, the evidence largely stems from research station experiments rather than commercial farm settings, which tend to have additional constraints on management decisions. The few on‐farm studies available suggest soil health practices are equivocal for forage production and environmental outcomes but are limited in geographic representation and scope, making extrapolation nationwide problematic. If there are nearly 2 acres of tillable land per cow and approximately 9 million cows, then we can estimate that there are roughly 18 million acres used for dairy forage production (Ishler, 2023; USDA‐NASS, 2022). Considering this number, adopting optimal production systems will have substantial environmental benefits. To foster widespread adoption of these practices, large‐scale, long‐term research on commercial dairy farms across diverse regions is crucial. We identify below the most important areas for future research.

### 8.1 Diverse geographic areas

Much of the on‐farm research has been concentrated in the Northeast and upper Midwest (see Section 5). However, five of the top 10 states for milk production in the United States in 2023 were California, Texas, New Mexico, Idaho, and Washington. From our survey of the literature, we identified two studies on commercial dairy farms in California comparing soil health management practices and quantifying GHG emissions, one study comparing practices and water quality outcomes in California, and one study comparing practices and GHG emissions on a dairy farm in Washington (Table 1). Furthermore, we did not identify any on‐farm research aimed at comparing soil health management practices to improve water use efficiency in any state other than Minnesota.

### 8.2 On‐farm research

Commercial dairy farms have focused on optimizing production of forages because they need to feed their animals; they often operate under different constraints than traditional plot studies at research stations. The fields on these farms have a long history of manure applications and have more topographic and hydrologic variability than most research plots. These factors will affect what soil health practices can be adopted, how they are adopted, and their effectiveness. Locally relevant research, at scales applicable to farm fields, and ongoing practices to evaluate the combinations of agronomic practices and manure management are needed. These studies should last more than a single growing season because the change in management may take several years to become evident (W. E. Jokela et al., 2009; Meals et al., 2010; T. O. West & Six, 2007; Wood & Bowman, 2021).

### 8.3 Systems approaches

No individual practice can maximize water use, improve water quality, and reduce GHG emissions because there are trade‐offs with each practice (Kong et al., 2009; Mitchell et al., 2015). Identifying the right combinations of practices for the soil type, climate, landscape, and operation type will provide optimal benefits. For example, no‐till systems with cover crops can reduce erosion and nutrient runoff, but managing nitrogen and crop residue during spring termination is crucial to avoid increased GHG emissions. Similarly, it is important to understand how these practices affect the whole field. For example, if these practices improve infiltration, they may increase water availability in water‐limited parts of the field and prevent ponding from runoff in other parts of the field. Redistribution of water on the landscape due to improvements in soil structure from no‐till practices should significantly impact GHG emissions, water infiltration and availability, and erosion.

### 8.4 Integrating new technologies

A wide variety of technologies are being explored to make better use of nutrients by limiting NH_3_ and GHG emissions (Hou et al., 2017; Ti et al., 2019; Cao et al., 2020) or removing excess phosphorus (He et al., 2019), especially during manure storage on farms. Advancements in manure technologies that create more economically transportable products or those designed to slowly release N and P can enhance the management of nutrient cycling and GHG emissions in forage production fields, but few studies have evaluated these new products in the field (Baxter et al., 2024; Seidel et al., 2017). Similarly, applying nutrients using precision agriculture practices is underexplored for manure but could reduce GHG emissions and improve water quality outcomes.

### 8.5 Improving water use efficiency

As dairy cow numbers continue to grow in the semiarid regions of the United States, farmers will need to optimize water use to grow forage. Some farmers have turned to growing forages with high water stress tolerance such as sorghum or triticale, but more intervention will be needed as water availability continues to decrease. Integration of a systems approach to include soil health management practices, water stress tolerant forages, and alternative irrigation strategies including drip irrigation should be explored. Research should specifically focus on identifying systems that reduce evapotranspiration, improve SOC to increase water holding capacity, and reduce erosion from dairies located in water limited regions.

### 8.6 Reducing GHG emissions

Identifying forage production systems to implement on dairy farms that reduce GHG emissions is an industry‐wide initiative. Research should specifically focus on identifying soil health management systems and application methods that reduce GHG emissions and increase SOC in the regions where the preponderance of dairy farms are located. There can also be tradeoffs based on management decisions. For example, incorporating or injecting manure can almost eliminate NH_3_ volatilization, which is associated with indirect N_2_O emissions, but can increase direct N_2_O emissions (Duncan et al., 2017).

### 8.7 Improving water quality

Leveraging soil health management practices to reduce runoff and leaching has received the most attention in humid regions of the United States, particularly in Minnesota, New York, Pennsylvania, and Wisconsin. However, farmers in other parts of the United States are increasingly being pressured to reduce runoff and leaching and have little to no scientific results to help guide their conservation planning, other than building and following nutrient management plans or extrapolating results from research conducted elsewhere. Research should specifically focus on identifying soil health management systems in the Western United States that reduce N and P losses through runoff and leaching.

## 9 CONCLUSION

The focus of this paper was on sustainable dairy forage production in the United States; however, the international dairy industry shares similar objectives and approaches. For example, researchers in Ireland are conducting on‐farm assessments to evaluate indicators of sustainability (Ryan et al., 2016). In Canada, researchers are experimenting with perennial forages to identify rotations that optimize farm economics and environmental outcomes (Ouellet et al., 2021). In New Zealand, a country with pasture‐based grazing systems, researchers are working to identify optimal grazing rotations and forages that support GHG reductions and improved water quality (DairyNZ, 2024). A review at the global scale found limited evidence for differences in nutrient losses between confined and grazed systems (McDowell et al., 2022).

These global efforts highlight the shared challenges and opportunities in adapting forage production systems to balance productivity, environmental stewardship, and economic feasibility. Forage production systems should be optimized to meet farm needs, effective at meeting environmental goals, and practical to implement for farmers across the types of US dairy operations. Improving environmental outcomes is a priority, and creating solutions that are economically feasible is a requirement for long‐term success. Combined with plot‐scale and off‐dairy research, future research on commercial dairy farms will provide the needed insights into reducing the perceived risk of adoption and developing comprehensive strategies to enhance environmental sustainability. This will ensure that the US dairy industry can thrive economically while minimizing its environmental footprint.

## AUTHOR CONTRIBUTIONS

**Mara L. Cloutier**: Conceptualization; data curation; visualization; writing—original draft; writing—review and editing. **Daniel Liptzin**: Conceptualization; data curation; visualization; writing—original draft; writing—review and editing. **Adolfo Coyotl**: Conceptualization; writing—original draft. **Abigail E. Baxter**: Conceptualization; writing—original draft. **Cristine L. S. Morgan**: Conceptualization; funding acquisition; writing—review and editing.

## CONFLICT OF INTEREST STATEMENT

The authors declare no conflicts of interest.
